# Exploring Technological, Safety and Probiotic Properties of *Enterococcus* Strains: Impact on Rheological Parameters in Fermented Milk

**DOI:** 10.3390/foods13040586

**Published:** 2024-02-15

**Authors:** Souraya Sakoui, Reda Derdak, Oana Lelia Pop, Dan Cristian Vodnar, Fatimazahra Jouga, Bernadette-Emőke Teleky, Boutaina Addoum, Elemér Simon, Ramona Suharoschi, Abdelaziz Soukri, Bouchra El Khalfi

**Affiliations:** 1 Laboratory of Physiopathology, Molecular Genetics & Biotechnology, Faculty of Sciences Ain Chock, Health and Biotechnology Research Centre, Hassan II University of Casablanca, Maarif B.P 5366, Casablanca 20000, Morocco; sakouisouraya@gmail.com (S.S.); reda.derdakk@gmail.com (R.D.); jougafatimazahra@gmail.com (F.J.); a_soukri@hotmail.com (A.S.); 2 Department of Biology, Faculty of Sciences El Jadida, Chouaïb Doukkali University, B.P 20, El Jadida 24000, Morocco; 3 Department of Food Science, University of Agricultural Science and Veterinary Medicine, Calea Mănăștur 3–5, 400372 Cluj-Napoca, Romania; oana.pop@usamvcluj.ro (O.L.P.); dan.vodnar@usamvcluj.ro (D.C.V.); bernadette.teleky@usamvcluj.ro (B.-E.T.); simon.elemer@usamvcluj.ro (E.S.); 4 Molecular Nutrition and Proteomics Lab, CDS3, Life Science Institute, University of Agricultural Science and Veterinary Medicine, Calea Mănăștur 3–5, 400372 Cluj-Napoca, Romania; 5 Food Biotechnology and Molecular Gastronomy, CDS7, Life Science Institute, University of Agricultural Science and Veterinary Medicine, Calea Mănăștur 3–5, 400372 Cluj-Napoca, Romania; 6 Biology and Medical Research Unit, Centre National de l’Energie, des Sciences et des Techniques Nucléaires, Rabat 10001, Morocco; addoumboutaina72@gmail.com

**Keywords:** bat guano, *Enterococcus*, technological properties, milk fermentation

## Abstract

Enterococci, known for their resilience, are commonly found in food, the environment, and the gastrointestinal tracts of humans and animals. In recent research, six strains of enterococcus were isolated from bat guano. These include *Enterococcus mundtii* SRBG1, *Enterococcus gallinarum* SRBG3, *Enterococcus faecium* SRBG2, *Enterococcus casseliflavus* EC1, and *Enterococcus devriesei* CAU 1344. Identification was done using 16S DNA analysis. Each strain underwent evaluation for its technological properties (such as tolerances to various NaCl concentrations and temperatures, as well as amylolytic, β-galactosidase, lipolytic, and proteolytic activities, and EPS production) and selected probiotic properties (including safety profile, resistance to 0.3 percent bile salts and gastric juice with a pH of 2.5, lysozyme tolerance, and antibacterial and antibiofilm activities against four foodborne pathogens). The results were analyzed using Principal Component Analysis. This analysis revealed that *E. mundtii* SRBG1 and *E. gallinarum* SRBG3, followed by *E. faecium* SRBG2, were most closely associated with a broad range of technological characteristics and were subsequently used for fermenting skimmed milk. The rheological properties of the samples indicated a shear-thinning or non-Newtonian behavior. Furthermore, during storage of the fermented milk at 4 °C over periods of 1, 7, 14, and 21 days, there were no significant changes in bacterial count (at around 7 log_10_ CFU/mL) and pH when fermented with the three evaluated strains.

## 1. Introduction

*Enterococcus* bacteria, colloquially known as enterococci and previously classified under “fecal” or Lancefield group D streptococci, are distinguished as Gram-positive, catalase-negative cocci [1,2]. Current research identifies approximately twenty distinct species within the *Enterococcus* genus, with *E. faecium* and *E. faecalis* emerging as the predominant species, especially in food-related environments [3]. These bacteria exhibit a wide distribution across various food types, notably in fruits, vegetables, and predominantly in animal-derived products such as dairy [2,4,5,6,7]. Additionally, they are prevalent in the gastrointestinal tracts of mammals, including bats [7]. Their ability to endure and adapt to a myriad of environmental conditions—ranging from low pH levels and high temperatures to elevated salinity—facilitates their survival and proliferation in diverse settings beyond the gastrointestinal tract, including soil, aquatic environments, and on plant surfaces. This adaptability is crucial for their capability to contaminate and multiply within raw food items like milk and meat throughout the fermentation process [7].

*Enterococci* are used in a wide range of applications throughout many distinct processes. For example, they are crucial for the ripening and flavor development of a variety of foods, including cheeses and sausages [5]. Their primary function in dairy products is to help the products’ organoleptic features develop as they grow and mature. Additionally, these bacteria have been used as probiotics to cure animal and human gastroenteritis and to enhance the microbial balance of the intestine.

A wide range of *Enterococcus* strains exhibit significant biochemical and biotechnological attributes, such as the capacity to metabolize citrate and perform a variety of metabolic functions, including proteolytic, lipolytic, and esterolytic activities. These characteristics are instrumental in conferring technological benefits in the realm of food processing [5,8,9]. Moreover, enterococci play a pivotal role in augmenting food safety and extending shelf life, attributed to their production of antimicrobial agents like lactic acid, hydrogen peroxide, and bacteriocins [5,8,9]. 

On the other hand, some Enterococcal strains could have the ability to carry plasmid-mediated resistance genes, which can be passed from one bacterial species to another and reduce a bacterium’s susceptibility to common antibiotics. The enterococci that carry these plasmid-mediated genes have been linked to the development of Vancomycin-Resistant Enterococci (VRE), which represent a challenge in clinical settings [10].

This study project aims to isolate *Enterococcus* strains from a relatively unexplored microflora domain, namely, bat guano, with the objective of elucidating their technological attributes. These attributes encompass a spectrum of features, such as resilience to diverse salinity and temperature conditions, alongside capabilities like amylolytic, proteolytic, β-galactosidase, and lipolytic activities, as well as exopolysaccharides (EPS) production. The study also assesses the strains’ safety profile, specifically their hemolytic activity, gelatinase capacity, and vancomycin resistance, and their resistance to various gastrointestinal challenges, including lysozyme, bile salts, and gastric juice. Utilizing Principal Component Analysis (PCA), the investigation aims to identify the most apt strains for deployment as starter cultures in the fermentation of skimmed milk. Additionally, the research examines the influence of EPS-producing strains on the rheological aspects, bacterial growth, and pH levels in fermented milk. By comprehensively analyzing these properties in the context of their origin and species, the study anticipates facilitating the preliminary selection of Enterococcus strains suitable for incorporation as starter agents in the food fermentation industry.

## 2. Materials and Methods

### 2.1. Chemical and Reagents

The following media and reagents were acquired from various suppliers: De Man, Rogosa and Sharpe (MRS), Mueller–Hinton (MH), Tryptic Soy Broth (TSB), and Brain Heart Infusion (BHI) broth were sourced from Biokar Diagnostics (Beauvais, France). Phenol and crystal violet were procured from Merck (Darmstadt, Germany). Additionally, a range of substances including lysozyme, pepsin, sodium chloride (NaCl), bile salts, starch, Arabic gum, and sucrose were obtained from Sigma-Aldrich (Saint Louis, MO, USA).

### 2.2. Isolation of Strains

To isolate the strains under study, 2 g of freshly collected bat guano from the “Kef Aziza” cave, located near the Boudnib region in Er-rachidia, Morocco, were suspended in a sodium chloride (NaCl) solution 0.9%. This was followed by a tenfold serial dilution in the same NaCl solution. From each dilution, 100 microliters were plated onto de Man, Rogosa and Sharpe (MRS) agar. These plates were then incubated at 37 °C for a period ranging from 24 to 72 h, enabling the enumeration and isolation of dominant bacterial colonies. Subsequently, distinct colonies were individually transferred to separate plates to establish pure cultures. These cultures were then placed in MRS broth with a 20% glycerol concentration and stored at −20 °C for future research purposes. The isolated strains were also characterized for their Gram-staining reaction and catalase production.

### 2.3. Molecular Identification

The extraction of bacterial DNA was performed using the Maxwell^®^ RSC Cell DNA Kit provided by Promega (Ref AS1370, Madison, WI, USA). The Polymerase Chain Reaction (PCR) was conducted using the extracted DNA. This PCR process followed the methodology previously described by [6]. The PCR mixture, with a total volume of 20 µL, consisted of approximately 100 ng of genomic DNA, 10 mmol/L of deoxynucleotide triphosphates (dNTPs), 5X PCR buffer, 25 mmol/L MgCl_2_, 10 mmol/L of each primer, and 5U of Taq polymerase (Promega). The amplification was carried out in a DLAB TC1000-G thermocycler. The PCR thermal profile was set as follows: an initial denaturation at 95 °C for 2 min, followed by 35 cycles of denaturation at 95 °C for 40 s, annealing at 55 °C for 40 s, and extension at 72 °C for 1 min; this was concluded with a final extension at 72 °C for 5 min and then held at 4 °C. The PCR products were purified using the ExoSAP-IT Purification Kit (GE Healthcare, Chalfont St. Giles, UK), and sequencing was performed using the BigDye Terminator Kit version 1.0 (Applied Biosystems, Darmstadt, UK). The chromatograms were read and corrected using the FinchTv software (1.4) and then introduced into the Geneious Prime software (Version 2022.2.1) where the sequences were aligned using BLAST: https://blast.ncbi.nlm.nih.gov/Blast.cgi (accessed on 20 May 2019).

The Neighbor-Joining approach was used to infer the evolutionary history. The evolutionary distances are expressed in base substitutions per site and were calculated using the Maximum Composite Likelihood approach. There were six nucleotide sequences in this investigation. For every sequence pair, all ambiguous positions were eliminated (pairwise deletion option). The final dataset contained 1145 locations in total. Evolutionary analyses were performed using MEGA11 software (11.0.13) [11].

### 2.4. Probiotic Assessment

#### 2.4.1. Safety Profile

The assessment of hemolytic activity was conducted based on the procedure outlined by [12]. For this test, blood agar plates (provided by Sigma Aldrich), enriched with 5% (*v*/*v*) blood, were inoculated with a 1% concentration of the lactic acid bacteria (LAB) strain. This was followed by incubation at 37 °C in aerobic conditions for a duration of 48 h. Post-incubation, the plates were examined to identify the presence or absence of clear zones (halos) surrounding the bacterial colonies, indicating hemolytic activity.

In parallel, the evaluation of gelatinase activity was performed in accordance with the methodology described by [6]. For this assay, 10 µL of the overnight bacterial culture was applied onto Luria–Bertani (LB) agar plates, which were supplemented with 5% (*w*/*v*) gelatin. The plates were incubated at two different temperatures: first at 28 °C for 72 h, and then at 37 °C for 48 h. After incubation, the plates were inspected for the presence or absence of clear zones around the colonies, indicative of gelatinase activity.

The strains’ development in the presence of vancomycin was examined as described by [13]. Activated cultures were grown on slopes, and the growth was cleaned from the surface using saline solution (NaCl 0.9%). Cell suspensions were used to inoculate Mueller–Hinton agar plates and the antibiotic was administered in discs containing 30 mg of vancomycin.

#### 2.4.2. Bile Salts and Gastric Juice Tolerance

The resistance of probiotic bacteria to bile salts and gastric juice was assessed following the protocol established by [6]. Briefly, bacterial strains at a concentration of 10^8^ CFU (colony forming units) per mL were inoculated into MRS broth. This was done for both the control group and the experimental groups, with the latter including MRS broth supplemented with either 0.3% (*w*/*v*) bile salts or a simulated gastric juice mixture (consisting of 0.5% sodium chloride and 0.3% pepsin, adjusted to a pH of 2.5). The inoculated broths were then incubated at 37 °C for 4 h. Following the incubation period, viable cell counts were conducted on MRS agar at 0, 1, 2, and 4 h. The results were quantified as a percentage of viability relative to the control, calculated using the following equation (Equation (1)):

(1) $$\%\;of\;tolerance:\frac{Number\;of\;viable\;cells}{Number\;of\;cells\;in\;control}\times 100$$

#### 2.4.3. Resistance to Lysozyme

The lysozyme resistance of the evaluated strains was determined using a modified version of the method described by [14]. The bacterial strains (10^8^ CFU/mL) were cultured in MRS broth, which was supplemented with varying concentrations of lysozyme (100, 200, and 500 mg/L). These cultures were incubated at 37 °C, and bacterial counts were performed at 0, 1, and 4 h post-inoculation. The survival of the bacteria in the presence of lysozyme was quantified and expressed as a percentage, calculated using Equation (2):

(2) Survival rate%=CtC0×100

where C_0_ is the CFU/mL at time 0, and C_t_ is the CFU/mL at 1 h and 4 h.

### 2.5. Antibacterial Activity

The antibacterial activity of the probiotic strains was assessed against several food-borne pathogens: *Staphylococcus aureus* ATCC 25923, *Escherichia coli* ATCC 8739, *Listeria monocytogenes* ATCC 19115, and *Enterococcus faecalis* ATCC 29212. This evaluation employed the well diffusion method, as previously described by [15]. The probiotic strains were incubated at 37 °C for 24 h. Post-incubation, the cell-free supernatant (CFS) was collected by centrifuging the culture at 8000× *g* for 10 min.

The pathogenic bacteria were first cultured in appropriate broths—Mueller–Hinton (MH), Tryptic Soy Broth (TSB), or Brain Heart Infusion (BHI) broth. These cultures were then incorporated into the same medium, now solidified with 1.5% agar. After the agar solidified, 50 µL of the previously obtained CFS was added to each designated well on the agar plates. These plates were then incubated for an additional 24 h at 37 °C to observe the inhibition zones, which indicate antibacterial activity.

### 2.6. Antibiofilm Activity

The assessment of antibiofilm activity was conducted using the method described by [16]. Initially, the overnight cultures of the strains were centrifuged at 8000× *g* for 24 h, and the resulting supernatant was collected for biofilm inhibition evaluation. Given that all the pathogenic bacteria tested in the antibacterial assay exhibited significant biofilm formation capabilities, they were chosen to determine the antibiofilm efficacy of the lactic acid bacteria.

In the assay, 50 µL of a 10^5^ CFU/mL bacterial culture and an equal volume (50 µL) of CFS were introduced into each well of a 96-well microplate. The plate was then incubated at 37 °C for 24 h. Post-incubation, the contents of each well were discarded, and the wells were washed three times with 300 µL of phosphate-buffered saline (PBS) at pH 7.4. The microplate was air-dried at 37 °C for 30 min. Subsequently, crystal violet (1 g/L) was applied to stain the biofilm-forming cells. After staining, the wells were rinsed three times with cold distilled water. Then, 300 µL of a dissolving solution composed of 30% methanol and 10% acetic acid were added to the wells. The absorbance, indicative of biofilm formation, was measured at 570 nm using a Synergy HT microplate reader (BioTek Instruments, Winoosku, VT, USA). The results were quantified according to Equation (3), which typically involves calculating the biofilm biomass based on the absorbance values:

(3) % of biofilm inhibition=1−AtreatmentAcontrol×100

where A_treatment_ is the absorbance of the treated culture with CFS, and A_control_ is the absorbance of the untreated culture.

### 2.7. Physiological and Biochemical Properties

#### 2.7.1. Growth under Different Concentrations of NaCl

The strains were initially grown in MRS broth and incubated at 37 °C for 24 h. Following this incubation, they were centrifuged at 8000× *g* for 10 min. The resulting pellet was collected, washed with phosphate-buffered saline (PBS) at pH 7.4, and then resuspended in the same buffer to achieve a concentration of 10^6^ CFU/mL. This bacterial suspension was subsequently inoculated into an MRS broth that had been supplemented with varying concentrations of NaCl (2, 6, and 10 g/100 mL) and incubated again at 37 °C for 24 h.

After incubation, the cultures underwent a serial dilution process and were then plated on MRS agar plates. The growth ability under these different NaCl concentrations was quantified and presented as the logarithm of colony-forming units per milliliter (log CFU/mL). A culture in unmodified MRS broth served as the control for comparison.

#### 2.7.2. Strains’ Growth under Different Temperatures

The methodology used to assess the temperature tolerance of the strains was adapted from the procedure outlined by [17]. The strains were cultivated in MRS broth and subjected to incubation periods of 24 h at three distinct temperatures: 15 °C, 37 °C, and 50 °C. Following incubation, the cultures were spread onto MRS agar plates. The growth of the strains at these varying temperatures was quantified and reported as the logarithm of the colony-forming units per milliliter (log CFU/mL). This approach allows for a clear comparison of the strains’ growth capabilities under different thermal conditions.

#### 2.7.3. Enzymatic Activities

##### Amylolytic Activity

The evaluation of amylolytic activity was conducted in accordance with the method described by [18]. In this process, the strains were cultivated in a modified MRS broth to which 1% starch had been added. This broth was then inoculated with 2% of an overnight culture of the strains and incubated at 37 °C for 24 h. Following the incubation period, the culture was centrifuged at 8000× *g* for 10 min, and the resulting supernatant was collected for starch content analysis.

The determination of starch content was performed using iodine, a common reagent used for detecting the presence of starch. The absorbance of the iodine–starch complex was measured at 580 nm using a Synergy HT microplate reader (BioTek Instruments, Winoosku, VT, USA). The rate of starch hydrolysis, indicative of amylolytic activity, was quantified by following Equation (4). This equation typically involves calculating the decrease in starch concentration, as indicated by the absorbance readings, to determine the extent of starch breakdown by the bacterial amylases.

(4) Starch hydrolysis rate=A0−A1A0×100

where A_0_ represents the absorbance of the initial culture containing starch, and A_1_ is the absorbance after 24 h of incubation.

##### Proteolytic Activity

The evaluation of proteolytic activity in the strains was carried out using a method outlined by [19]. Initially, the strains were cultured in MRS broth and incubated at 37 °C for 24 h. Post-incubation, the cultures were centrifuged at 8000× *g* for 10 min at a temperature of 4 °C to separate the cells from the supernatant.

Following centrifugation, a sterile paper disc was impregnated with the collected supernatant. This disc was then placed onto a plate containing skimmed milk agar (prepared at a concentration of 1 g/100 mL). The agar plates were subsequently incubated at 37 °C for 24 h. The presence of a clear zone around the paper disc on the skimmed milk agar was interpreted as an indication of proteolytic activity. This clear zone results from the hydrolysis of milk proteins by proteolytic enzymes present in the supernatant, demonstrating the strain’s capacity to break down proteins.

##### Lipolytic Activity

The assessment of the lipolytic activity of the strains was carried out following the methodology outlined by [19]. The strains were first cultured in MRS broth that had been supplemented with 1% extra virgin olive oil and Arabic gum. This culture was then incubated at 37 °C for 24 h. After the incubation, aliquots of the culture were applied onto a modified MRS agar, which also contained 0.5–1% olive oil and Arabic gum. The appearance of clear zones around the colonies on this agar indicated lipolytic activity. These clear zones were formed due to the degradation of lipids (fats) in the agar by the lipase enzymes produced by the strains, demonstrating their ability to hydrolyze fats.

##### β-Galactosidase

The β-galactosidase activity of the strains was evaluated as described previously by [6]. The LAB strains were streaked into MRS agar that included 10 μL iso-propyl-thio-β-d-galactopyranoside solution and 60 μL of 5-bromo-4-chloro-3-indolyl-β-d-galactopyranoside. The plates were incubated at 37 °C for 42 h. Strains grown in MRS agar were used as the control.

#### 2.7.4. EPS Production

The production of exopolysaccharides (EPS) was evaluated using a slightly modified version of the protocol described by [20]. The initial screening for EPS-producing strains involved streaking an overnight culture onto MRS agar medium, where glucose was replaced with 10% sucrose. These plates were then incubated at 37 °C for 72 h. Strains that exhibited EPS production were further selected for the quantitative analysis of EPS.

In the quantification step, the selected strains were inoculated into MRS broth containing 10% sucrose and incubated at 37 °C for 72 h. Following this incubation period, the supernatant was obtained through centrifugation at 8000× *g* for 15 min. To precipitate the EPS, cold ethanol in a 2:1 ratio was added to this supernatant. The mixture was then incubated overnight at 4 °C. Post-incubation, the EPS was isolated by another round of centrifugation at 8000× *g* for 15 min. The carbohydrate content of the crude EPS extract was then determined using the phenol–sulfuric acidmin method, as described by [21]. This method is widely used for quantifying total carbohydrate content in a sample.

### 2.8. Fermentation of Milk Process and Rheological Parameters

The assessment of the fermentation capability of the selected strains was conducted in accordance with the method described by [12]. Skimmed milk at a concentration of 5% (*w*/*v*) was pasteurized at 100 °C for 5 min using a water bath. After cooling to 37 °C, the milk was aseptically inoculated with 10^8^ CFU/mL of the strain and then incubated at 37 °C for 24 h. Post-fermentation, any milk sample that exhibited coagulation was further analyzed for rheological properties. Non-fermented milk served as the control for these experiments.

For rheological measurements, an Anton Paar MCR 72 rheometer (Anton Paar, Graz, Austria), equipped with a Peltier plate–plate system (P-PTD 200/Air), was employed. The rheometer’s lower plate was set to maintain a temperature of 25 °C and a gap of 1 mm, while the upper plate featured a smooth parallel plate geometry with a diameter of 50 mm. Before commencing measurements, any excess sample was removed, and the sample was allowed to rest for one minute to reach thermal equilibrium. The viscosity of each sample was measured twice, with the shear rate linearly increasing from 5 to 300 1/s. This range of shear rates was chosen to mimic the intense shearing forces encountered during various industrial processes.

#### Bacterial Enumeration and pH Measurement

The milk samples fermented with various strains were chilled and then stored at 4 °C for a duration of three weeks. On days 1, 7, 14, and 21, samples were taken from each batch of fermented milk. These samples were diluted as necessary using a saline solution. Bacterial enumeration was subsequently conducted on MRS agar plates. The results of this bacterial counting were expressed in terms of colony-forming units per milliliter (CFU/mL). Additionally, the pH value of each milk sample was determined using a pH meter, providing an indication of the acidity level in each fermented milk sample over the storage period.

### 2.9. Statistical Analysis

All experiments were conducted in triplicate, and the outcomes have been reported as the mean ± standard deviation. The collected quantitative data served as the input for Principal Component Analysis (PCA), which was carried out using the XLSTAT software (2016) in Excel. For a comparative analysis of the mean values of pH and bacterial count, GraphPad Prism version 8.0.2 was utilized, employing the one-way ANOVA test for statistical evaluation.

## 3. Results and Discussion

### 3.1. Phenotypic and Molecular Identification 

A total of 25 lactic acid bacteria (LAB) strains were isolated, all of which were characterized as Gram-positive. Among these isolates, seven were identified as catalase-negative and cocci-shaped, with the exception of one isolate that was bacillus. The sequencing data for all isolates were visualized using FinchTV software and subsequently analyzed for identification with Geneious Prime Software. For the purposes of this study, only the *Enterococcus* strains were selected. These selected strains were identified as *Enterococcus mundtii* SRBG1, *Enterococcus gallinarum* SRBG3, *Enterococcus casseliflavus* EC1, *Enterococcus* sp., *Enterococcus faecium* and *Enterococcus devriesei* CAU1344.

The genetic sequences we obtained have been deposited in the GenBank database (https://www.ncbi.nlm.nih.gov/genbank/) (accessed on 18 January 2023), which is maintained by the National Center for Biotechnology Information (NCBI). Each of these sequences has been assigned a unique accession number by GenBank (Table 1). These accession numbers serve as unique identifiers to reference and retrieve the information associated with each sequence in the database, thus allowing traceability and the public consultation of our genetic data.

**Figure 1:**
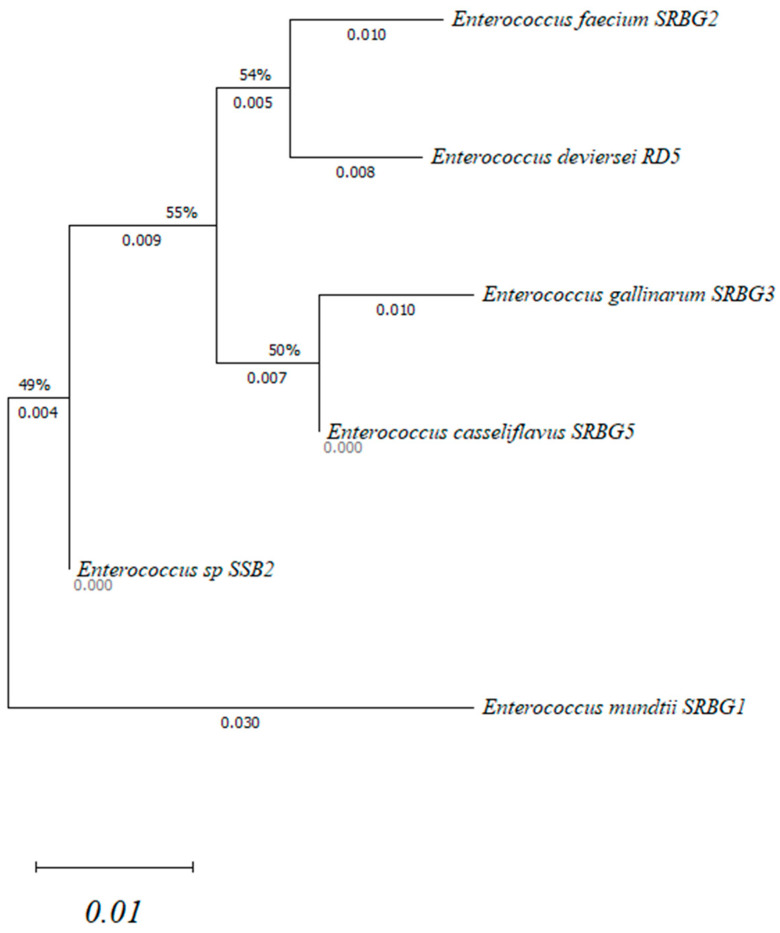
Neighbor-joining tree based on the 16S rRNA gene.

In order to understand the phylogenetic relationships within the genus *Enterococcus*, we constructed a phylogenetic tree based on the 16S rRNA sequences. The clustal w program was used to align the resultant sequences. Neighbor-joining was used to reconstruct the phylogenetic tree (Figure 1). A bootstrap analysis based on the 1145 position was carried out. The phylogenetic analysis was conducted using the MEGA11 software (11.0.13) program [22]. 

The 16S rRNA gene sequence analysis showed three branches. The results of the determination of the disparity degree between the studied isolates and their classification according to the phylogenetic tree are illustrated in Figure 1. The first group contained four strains, which were *Enterococcus faecium* SRBG2, *Enterococcus deviersei* RD5, *Enterococcus gallinarum* SRBG3 and *Enterococcus casseliflavus* SRBG5. The second and the third groups included 1 *Enterococcus* strain each (*Enterococcus* sp. SSB2 and *Enterococcus mundtii* SRBG1) (Figure 1). The 16S rRNA gene is highly helpful in differentiating between the major enterococci species groupings, which include *E. avium*, *E. casseliflavus*, *E. cecorum*, *E. faecalis*, and *E. faecium*. However, it is not able to distinguish between closely related species. As an exemple, Devresei et al. (2002) reported that the species group *E. faecium*, which includes *E. hirae*, *E. durans*, *E. villorum*, *E. mundtii*, and *E. ratti*, have 98.8–99.7% similarities [22].

### 3.2. Probiotic Assessment

#### 3.2.1. Safety Profile

The results show that the bacteria tested did not exhibit hemolytic activity, as evidenced by the lack of halos around the colonies on blood agar plates. Additionally, no gelatinase activity was observed, which was marked by the absence of halos around colonies on Luria–Bertani (LB) agar supplemented with 5% (*w*/*v*) gelatin.

The absence of hemolytic activity in these bacteria suggests they lack the ability to lyse red blood cells. Hemolysis is typically a characteristic of certain pathogenic bacteria, in which the release of hemolytic enzymes leads to the disintegration of red blood cells, contributing to the pathogenicity of the organism [12]. Therefore, the lack of hemolytic activity in the bacteria we tested may indicate they are non-pathogenic in this regard, or lack the enzymes required for such activity.

Furthermore, the absence of gelatinase activity indicates that these bacteria do not produce enzymes capable of degrading gelatin. Gelatinases usually facilitate the breakdown of gelatin, a product derived from collagen, which is a structural protein in various tissues [6]. The presence of gelatinase activity can be harmful in certain situations, as it might lead to the degradation of the extracellular matrix and host tissues. In our study, the lack of gelatinase activity in the bacteria is seen as a beneficial attribute.

This suggests that the bacteria under study are not involved in the degradation of gelatin or collagen-related proteins. In applications like food production or biotechnological processes, bacterial gelatinase activity can cause the undesired degradation of products. Consequently, the absence of gelatinase production in these bacteria might be advantageous in scenarios where maintaining the integrity of gelatin-based materials or collagen-containing substances is crucial.

An optimal probiotic strain should have no virulence factors or developed antibiotic resistance. Enterococci may exhibit vancomycin resistance, which is a potentially harmful feature. Antibiotic resistance, which can be intrinsic or acquired, makes enterococci efficient opportunists in nosocomial infections. Vancomycin-resistant enterococci (VRE) are potentially the most serious threat to human clinical infections in terms of acquired antibiotic resistance [5]. In the present study, all the tested strains were sensitive to vancomycin (30 mg) with an inhibition zone ≥12 mm.

While our bacteria did not exhibit hemolytic or gelatinase activity, nor did they show resistance to vancomycin, it is crucial to investigate deeper into this aspect.

The security profile of probiotics, particularly in terms of virulence factors such as toxins, extracellular enzymes, and cytolysin, necessitates careful consideration before their use in food. A thorough evaluation of these characteristics is required to reduce potential hazards related with the introduction of probiotics into the human microbiome. Screening for virulence factors becomes an important stage in the evaluation process, ensuring that only strains with acceptable properties are chosen for usage.

#### 3.2.2. Bile Salts, Gastric Juice and Lysozyme Tolerance

**Figure 2:**
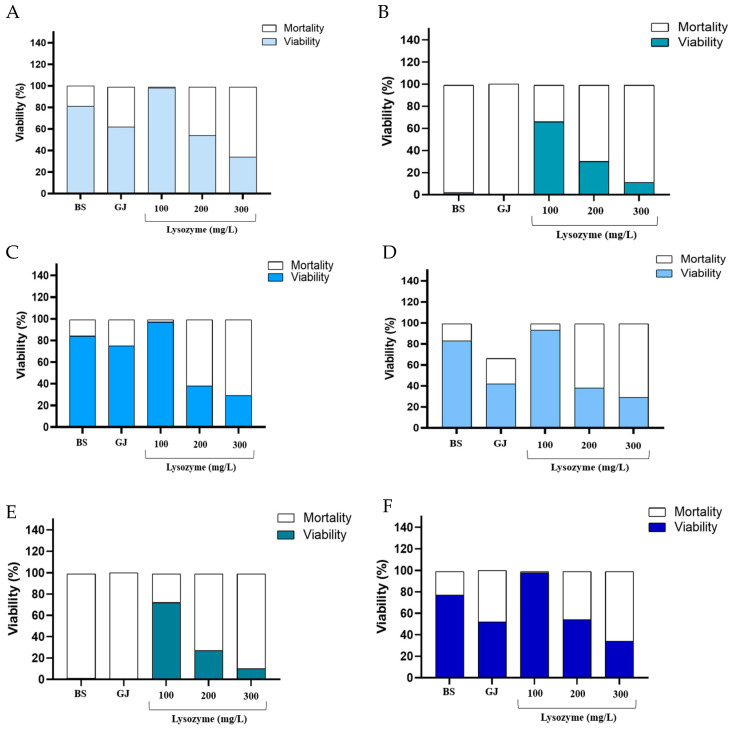
The viability percentages of (**A**) *E. mundtii* SRBG1, (**B**) *E. casseliflavus* EC1, (**C**) *E. gallinarum* SRBG3, (**D**) *E.* sp., (**E**) *E. devriesei* CAU10344 and (**F**) *E. faecium* SRBG2, under bile salts, gastric juice and different concentrations of lysozyme.

Probiotic bacteria must possess mechanisms to withstand the harsh conditions of the digestive tract, such as the high concentrations of bile salts (ranging from 0.3 to 2%) and low pH levels in stomach and gastric juices, to ensure they reach the small intestine in a viable and active state. These extreme conditions can potentially damage cell membranes, impeding the probiotics’ effectiveness [23]. Our study, illustrated in Figure 2, evaluates the tolerance of isolates to gastric juice and bile salts. After 4 h of exposure at 37 °C, most isolates demonstrated a significant survival rate, with percentages between 77.03 and 84.57% for bile salts and 42.85 and 75.71% for gastric juice. However, *E.* sp. and *E. devriesei* CAU10344 exhibited markedly lower viability in bile salts (2.92% and 1.32%, respectively) and did not survive in gastric juice. In contrast, Özkan, E.R. et al (2021) reported that *E. faecium* isolated from artisanal goatskin showed survival rates of 85.27–87.47% in 1% bile salts [23]. Hajikhani et al. (2021), studying bacteria from white cheese, noted that bile salt tolerance varied depending on the bacterial source [24]. Sun et al. (2010) found that *Enterococcus faecium* SF68 retained 56% viability after 60 min in simulated gastric juice [25]. Similarly, Bhardwaj et al. (2010) observed that *E. faecium* KH24 had survival rates of 40% and 60% when subjected to gastric juice at pH 2.5 and pH 3.0, respectively [26].

Lysozyme, present in human saliva, acts as an initial barrier for probiotic bacteria entering the gastrointestinal tract [27]. In our study, all tested strains showed the ability to resist various concentrations of lysozyme (100, 200, and 500 mg/L), with resistance rates ranging from 66.26% to 98.78%, 27.09% to 54.35%, and 10.05% to 34.71%, respectively. Singhal et al. (2019) reported that *Enterococcus* strains isolated from the Rhizosphere grew in 100 mg/L lysozyme with viability percentages ranging from 75% to 80% after 2 h of exposure [27].

Indeed, this indicates the existence of common pathways in bacterial responses to various stresses. Since, our strains showed the capacity to produce EPSs, these exocellular polymers have a significant impact on bacterial surface characteristics and serve as a protective coating against environmental conditions. Accordingly, it has been shown that bile can cause the synthesis of exopolysaccharides, most likely as a bile defense mechanism [28]. The presence of EPS increased tolerance towards nisin and lysozyme, but not to other antimicrobials (penicillin G and vancomycin).

#### 3.2.3. Antibacterial Activity

**Figure 3:**
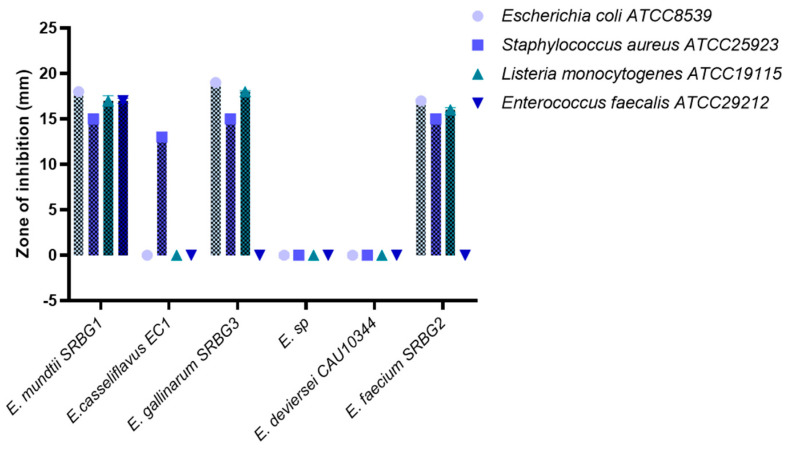
Antibacterial activity of the selected lactic acid bacteria against pathogenic ones.

Lactic acid bacteria (LAB) are recognized for enhancing food safety and quality by inhibiting spoilage and pathogenic microbes [29]. Notably, only a select number of enterocin-producing *Enterococci* strains have been identified to exhibit significant inhibitory effects against both bacteria and fungi [30]. In our study, each strain tested demonstrated inhibitory activity against all the pathogenic strains assessed, as shown in Figure 3. The most pronounced inhibitory activity was observed in *E. mundtii* SRBG1, followed by *E. gallinarum* SRBG3 and *E. faecium* SRBG2. However, *E. devriesei* CAU10344 did not show any inhibitory effects on the pathogenic strains.

Ben Braïek et al. (2018) reported that *Enterococcus lactis*, recently isolated from shrimp, displayed significant inhibitor activity [30]. This activity was retained even after heating the cell-free supernatant at 60 °C for 30 min and 100 °C for 15 min, and following a pH adjustment from 2 to 10. Similarly, Nami et al. (2019) found that all enterococcus strains they isolated from artisanal dairy products inhibited a broad range of pathogenic strains, including *Bacillus subtilis*, *Staphylococcus aureus*, *Listeria monocytogenes*, *Klebsiella pneumoniae*, *Yersinia enterocolitica*, *Escherichia coli*, and *Shigella flexneri* [3].

By generating antimicrobial chemicals, reducing pH, competing with pathogens at binding sites, and competing with them in food, enterococci have a protective or therapeutic impact. Several previous studies have demonstrated the function of Enterococcus strains in preventing food-borne pathogens [3,30]. 

The common feature of most Enterococci probiotic strains is the ability of these bacteria to produce metabolites and various antimicrobial compounds, including small peptides, bacteriocins, EPSs and organic acids, such as butyric, acetic, and lactic acid [4].

#### 3.2.4. Antibiofilm Activity

**Figure 4:**
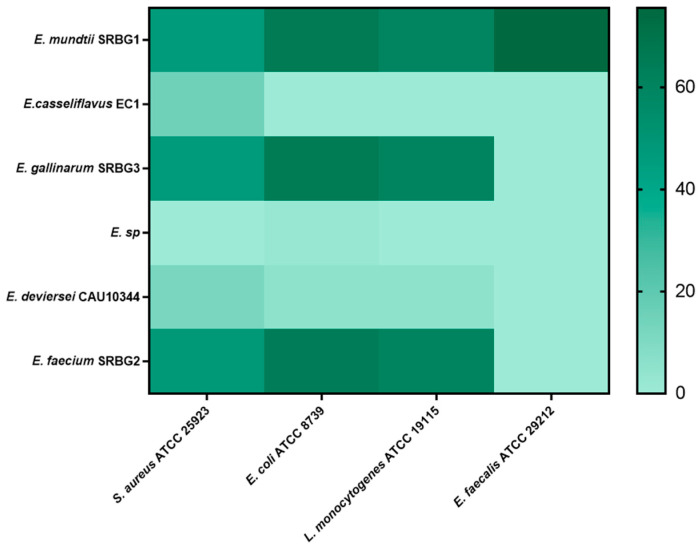
Heat map (percentage (%)) showing the antibiofilm activity of the selected strains against pathogenic ones.

Pathogens can withstand the demanding conditions of food preparation partly due to their ability to form biofilms on various surfaces. Lactic acid bacteria (LAB) are thus valuable not only for inhibiting pathogen growth, but also for their potential ability to prevent biofilm formation [31]. However, as illustrated in Figure 4, not all LAB strains studied were effective in inhibiting biofilm formation. *E. mundtii* SRBG1, in particular, was effective against all pathogenic bacteria tested.

For *S. aureus* ATCC 25923, the inhibition percentages of *E. mundtii* SRBG1, *E. gallinarum* SRBG3, and *E. faecium* SRBG2 were 46.74%, 46.48%, and 47.66%, respectively. Against *E. coli* ATCC 8739, the corresponding inhibition rates were 65.49%, 65%, and 64%. For *L. monocytogenes* ATCC 19115, the three isolates displayed similar inhibition percentages. Notably, *Enterococcus* spp. *E. devriesei* CAU10344 did not exhibit biofilm-inhibiting capabilities. Conversely, *E. mundtii* SRBG1 demonstrated higher efficacy against the biofilm of *E. faecalis* ATCC 29212, while the other isolates were ineffective against the *E. faecalis* biofilm.

In related research, Cui et al. (2018), who focused on LAB isolated from handmade milk cheese, found that one *E. faecium*, seven *Lactobacillus helveticus*, and three *Pediococcus acidilactici* strains showed antibiofilm activity against *S. aureus* CMCC26003 and *E. coli* CVCC230 [32].

### 3.3. Physiological and Biochemical Properties

#### 3.3.1. Growth under Different Concentrations of NaCl and Different Temperatures

**Figure 5:**
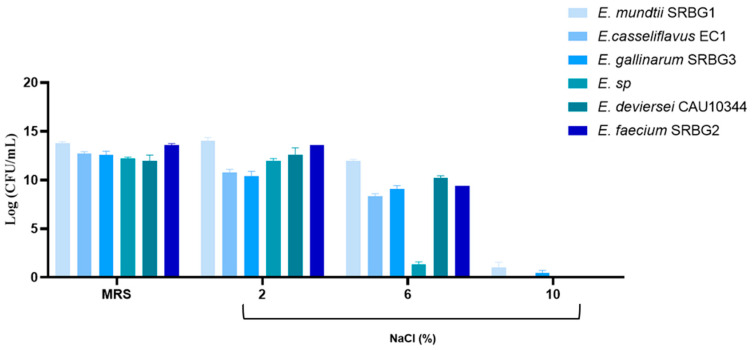
Growth of the selected strains under different concentrations of NaCl (2%, 6%, 10%).

In food processing, lactic acid bacteria (LAB) are subjected to various conditions, such as high salinity and fluctuating temperatures, particularly in the production of dairy products like cheese and sweet-cream butter. Our study found that, with the exception of *E.* sp., which grew at 6% NaCl with a 14% survival rate, all isolates exhibited resistance to 2 and 6% NaCl concentrations, with a range of 8.37–14.05 log CFU/mL. Only *E. mundtii* SRBG1 and *E. gallinarum* SRBG3 were able to grow in 10% NaCl after 24 h (Figure 5). Similarly, Morandi et al. (2013) reported that *E. lactis* could grow in the presence of 2, 4, and 6% NaCl [33]. Additionally, Ribeiro et al. (2014) found that, except for *E. faecalis* L3A1M6, none of the tested strains could grow above 2% NaCl, with one exhibiting resistance to as high as 10% NaCl. The strains’ tolerance to high salt concentrations equips them to handle the osmotic pressure in the gastrointestinal tract, maintaining osmotic balance under such conditions, which is crucial for their survival in food production [34]. 

**Figure 6:**
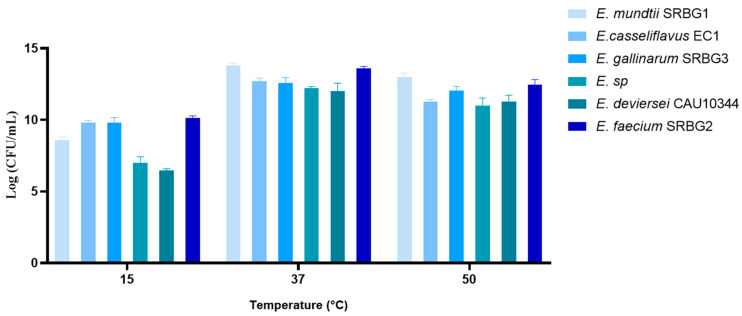
Growth of the selected strains under different temperatures (15 °C, 37 °C, 50 °C).

Concerning temperature, as depicted in Figure 6, variations impact bacterial survival and metabolism, thereby influencing their beneficial effects. All our strains could grow at 15 °C, 50 °C, and 37 °C, the latter being their optimum growth temperature. Morandi et al. (2013) also observed that all *Enterococcus* strains they studied could grow between 15 °C and 45 °C [33]. Our findings align with those of [17]. In cheese production, alongside pH and salt content, milk is treated at varying temperatures based on the cheese type. For instance, Emmental cheese requires milk to be heated to 50 °C, denaturing the enzymes from ruminant mammal stomachs (rennet), while feta cheese production involves temperatures of 14–16 °C. In the case of sweet-cream or salted butter production, the fermented milk is heated to between 14 and 20 °C after adding salt [35]. 

#### 3.3.2. Enzymatic Activities

LABs are used in food manufacturing due to the many beneficial compounds they produce, such as some enzymes (amylase, protease and lipase) that improve the structure and the organoleptic properties of food [34,36]. In our study, between six tested strains, only three showed an amylolytic activity: *E. mundtii* SRBG1, *E. gallinarum* SRBG2 and *E. faecium* SRBG2 (Table 2). While amylolytic activity is important in fermentation and improving starch-based food quality [37], most LAB does not possess this property [18]. 

Regarding the proteolytic activity, two out of six strains were able to hydrolyze casein, due to being positive for extracellular proteolytic activity (Table 2). Ribeiro et al. (2014) reported that the majority of enterococcus strains tested did not show an extracellular proteolytic activity, except for one isolate [17]. The results of the lipolytic activity show that none of the tested strain possessed lipolytic activity (Table 2). Anagnostopoulos et al. (2018) reported that enterococcus have a weak proteolytic and lipolytic activity [36]. Otherwise, the proteolytic and lipolytic activities are strain-dependent [34].

An essential characteristic of a probiotic strain is β-galactosidase activity. Because it hydrolyzes lactose into galactose and glucose, β-galactosidase is crucial in the management of lactose intolerance [3]. Three of the six strains in our research were able to hydrolyze lactose, which is a sign that β-galactosidase is being produced (Table 1). According to [38], only two of the isolated strains, *Enterococcus mundtii* QAUSD01 and *Enterococcus faecalis* QAUSD01, had β-galactosidase activity.

#### 3.3.3. EPS Production

In our research, only three strains were identified as EPS-producers, with *E. faecium* SRBG2, *E. gallinarum* SRBG3, and *E. mundtii* SRBG1 producing 0.134, 2.868, and 3.221 g/L of EPS, respectively, as shown in Table 2. The ability of LAB to produce exopolysaccharides (EPS) renders them valuable in the food industry, particularly for enhancing the texture and rheological properties of dairy products [17]. However, Ribeiro et al. (2014) noted that none of the *Enterococcus* strains in their study produced EPS [17]. Contrarily, Venkatesh et al. (2016) reported an EPS yield of 0.84 g/L from *E. faecalis* DU10 [39]. Similarly, a study focusing on EPS characterization and its stimulatory effect on *Bifidobacterium* spp. growth, conducted with *E. faecalis* EJRM152 isolated from human breast milk, observed a maximum EPS yield of 3.68 g/L.

### 3.4. Principal Component Analysis (PCA)

**Figure 7:**
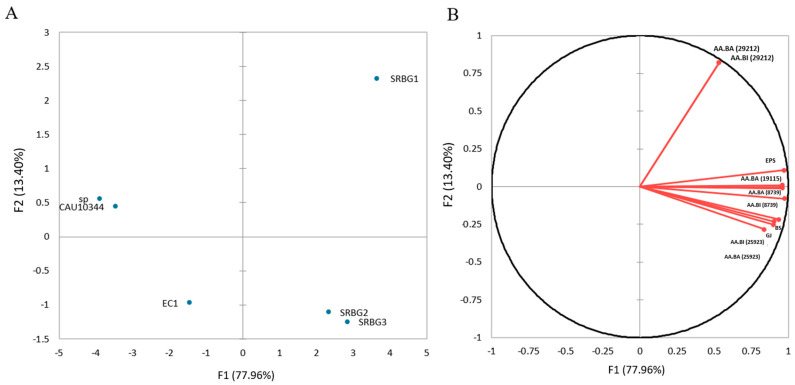
Distribution plots of observations (**A**) and variables (**B**) on the plane of the first and second principal components.

The Principal Component Analysis (PCA) in our study revealed that the first four principal components (PCs) collectively accounted for 91.36% of the total variation observed. Specifically, PC1 explained 77.96% of the variation, while PC2 accounted for 13.40% (Figure 7). As indicated in Table 3, each variable showed a strong association with both PC1 and PC2, suggesting that these components capture the key technological characteristics of the most promising isolates.

Figure 6 presents the scatter plots of these variables in the plane defined by the first two principal components. When mapping the LAB isolates onto the two-dimensional space formed by PC1 and PC2, three primary clusters were observed. The isolates situated in quadrants I and IV are particularly noteworthy because they correlate well with all the variables, indicating their significance. Among these, the isolates SRBG1, SRBG2, and SRBG3 emerged as the most promising candidates, showcasing superior technological properties.

### 3.5. Apparent Viscosity and Resistance to Shearing

**Figure 8:**
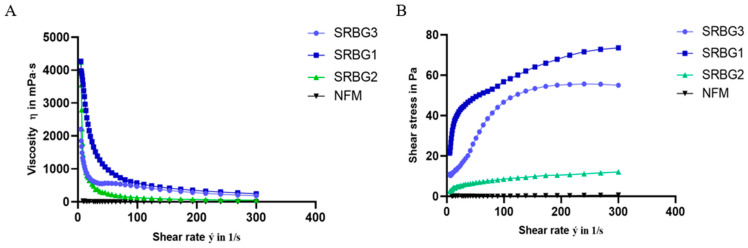
Apparent viscosity (**A**) and shear stability (**B**) of fermented milk with SRBG1, SRBG2 and SSRBG3 compared to the non-fermented milk (NFM).

The apparent viscosity and shear stability of milk fermented with strains SRBG1, SRBG2, and SRBG3 are depicted in Figure 8. The findings indicate that the viscosity of the fermented milk decreased with increasing shear rate, demonstrating that all samples exhibited non-Newtonian or shear-thinning behavior. This observation aligns with the findings reported by [40]. The flow curve’s shape varied depending on the strain, with all three tested strains exhibiting pseudoplastic behavior, corroborating the viscosity results (Figure 8B). 

Specifically, the shear stress in milk fermented with SRBG1 ranged from 21.347 to 69.869 Pa over a shear rate of 0 to 193 s^−1^. For milk fermented with SRBG3, the shear stress increased from 11.037 to 54.469 Pa at a shear rate range of 0 to 173 s^−1^, eventually plateauing at 55 Pa. In contrast, milk fermented with SRBG2 showed a more modest increase in shear stress, reaching only 12.128 Pa at 300 s^−1^. The pronounced shear stress plateau in milk fermented with SRBG1, which produced a higher EPS amount than SRBG2, could be due to interactions between the negatively charged EPS and the casein network, suggesting that a higher level of EPS production by a strain correlates with more pronounced stress-thinning behavior in the fermented milk flow curve.

In similar research, Surber et al. (2020) observed that milk fermented with the EPS-producing strain NCC1971 exhibited higher shear stress compared to milk fermented with the non-EPS-producing strain NCC2201 [41]. The shear stress of milk fermented with NCC1971 increased to a plateau around 70 Pa after reaching a maximum shear rate of 186 s^−1^, attributed to the resistance of EPS–bacterial cell bonds breaking under maximum treatment. However, the high shear rate stability of shear stress might also be linked to the water-binding capacity of polysaccharides, as noted by [42].

### 3.6. Bacterial Enumeration and pH Measurement

Table 4 presents the bacterial counts of strains SRBG1, SRBG2, and SRBG3 in fermented milk after 1, 7, 14, and 21 days of storage at 4 °C. Throughout the 21-day storage period, there was no statistically significant reduction (*p* > 0.05) in the viability of these three strains in skimmed milk at 4 °C. Additionally, all strains demonstrated effective acidification capabilities, as evidenced by a decrease in pH from the initial value of 6.7. These findings are in line with those reported by [19].

In a similar vein, Sarwar et al. (2019) noted that the increase in acidity observed in fermented milk during cold storage can be attributed to microbial activity and the enzymes produced during fermentation [43]. These enzymes facilitate the conversion of residual carbohydrates, primarily lactose, into lactic acid, CO_2_, and formic acid. This process contributes to the acidification of the milk over the course of storage.

## 4. Conclusions

The use of enterococci as probiotics and/or starter cultures in food preparation is a topic of discussion nowadays. In the present study, six *Enterococcus* strains (*Enterococcus mundtii* SRBG1, *Enterococcus faecium* SRBG2, *Enterococcus gallinarum* SRBG3, *Enterococcus casseliflavus* EC1, *Enterococcus* sp., and *Enterococcus devriesei* CAU1344) were newly isolated from bat guano. Each of these strains underwent extensive evaluation for their physiological biochemical properties and probiotic properties. This included assessments under various NaCl concentrations and temperatures, as well as evaluations of their amylolytic, β-galactosidase, lipolytic and proteolytic activities, and their EPS production. Additionally, their response to 0.3% bile salts, gastric juice at pH 2.5, different concentrations of lysozyme, and their antibacterial and antibiofilm activities were investigated. These tests revealed distinct responses from the strains to the different conditions and properties examined. On the other hand, the safety profile of these bacteria was also evaluated, such as their hemolytic and gelatinase activity and their vancomycin resistance.

Using the qualitative results obtained, Principal Component Analysis (PCA) was employed to identify the most promising candidates for use as starter cultures in milk fermentation. Notably, *Enterococcus mundtii* SRBG1, *Enterococcus gallinarum* SRBG3, and *Enterococcus faecium* SRBG2, which produced EPS at varying yields of 3.221, 2.868, and 0.134 mg/L, respectively, were selected for skimmed milk fermentation.

These three strains exhibited diverse behaviors in terms of apparent viscosity and shear rate response. A notable observation was that the greater the EPS production by a strain, the more resistance it exhibited to high shear rates. This suggests a correlation between EPS yield and the rheological properties of fermented milk. Given these findings, these strains appear to be promising for use in applications in the food industry, particularly due to their unique and beneficial technological characteristics.

The study’s results provide helpful information for guiding the choice of the right strains to utilize as protected cultures or starters for producing fermented foods that may have probiotic qualities. However, it is essential to continue to deepen the understanding of their mechanism of action and monitor their behavior in in vivo environments (as advised by FAO/WHO (2002)) to ensure their judicious and safe use in future probiotic applications [44].

## Figures and Tables

**Table 1:** Sequence’s accession number.

Strain	Accession Number
*Enterococcus mundtii* SRBG1	ON204234
*Enterococcus gallinarum* SRBG3	ON204236
*Enterococcus casseliflavus* SRBG5	OQ280989
*Enterococcus* sp. SSB2	OQ280990
*Enterococcus deviersei* RD5	OQ280993
*Enterococcus faecium* SRBG2	ON204235

**Table 2:** The enzyme activity of the selected strains and their ability to produce exopolysaccharides.

		*E. mundtii* SRBG1	*E.casseliflavus* EC1	*E. gallinarum* SRBG3	*E. faecium* SRBG2	*E.* sp.	*E. deviersei* CAU10344
Enzymes	Amylase	+	−	+	+	−	−
	Protease	−	−	+	+	−	−
	Lipase	−	−	−	−	−	−
	β-galactosidase	+	−	+	+	−	−
EPS production (g/L)		3.221 ± 0.3	−	2.868 ± 0.6	0.134 ± 0.2	−	−

**Table 3:** Correlation of variables to PCA analysis factors based on factor loadings.

	F1	F2	F3	F4	F5
Exopolysaccharide	0.938	0.012	0.047	0.004	0.000
Bile salts	0.708	0.080	0.209	0.001	0.001
Gastric juice	0.881	0.048	0.039	0.027	0.006
Lysozyme	0.821	0.055	0.110	0.013	0.001
Antibacterial activity (8739)	0.926	0.000	0.072	0.002	0.000
Antibacterial activity (25923)	0.809	0.063	0.124	0.000	0.004
Antibacterial activity (19115)	0.926	0.000	0.072	0.002	0.000
Antibacterial activity (29212)	0.284	0.672	0.045	0.000	0.000
Antibiofilm activity (8739)	0.915	0.000	0.083	0.001	0.000
Antibiofilm activity (25923)	0.954	0.006	0.017	0.018	0.005
Antibiofilm activity (19115)	0.910	0.000	0.088	0.001	0.000
Antibiofilm activity (29212)	0.284	0.672	0.045	0.000	0.000

**Table 4:** Bacterial count (log_10_ CFU/mL) and pH value of fermented milk with SRBG1, SRBG2 and SRBG3 after storage at 4 °C for 1, 7, 14 and 21 days.

	Storage (Day)	SRBG1	SRBG2	SRBG3
Bacterial count (log_10_ CFU/mL)	1	6.96 ± 0.10 ^a^	7.69 ± 0.24 ^c^	7.00 ± 0.71 ^e^
	7	7.03 ± 0.60 ^a^	7.60 ± 0.32 ^c^	7.34 ± 0.30 ^e^
	14	7.00 ± 0.12 ^a^	7.65 ± 0.11 ^c^	7.12 ± 0.63 ^e^
	21	7.12 ± 0.30 ^a^	7.67 ± 0.21 ^c^	7.15 ± 0.42 ^e^
pH measurement	1	4.34 ± 0.10 ^b^	5.10 ± 0.10 ^d^	5.24 ± 0.10 ^f^
	7	4.30 ± 0.00 ^b^	5.11 ± 0.20 ^d^	5.20 ± 0.00 ^f^
	14	4.26 ± 0.10 ^b^	5.07 ± 0.00 ^d^	5.16 ± 0.10 ^f^
	21	4.28 ± 0.12 ^b^	5.10 ± 0.10 ^d^	5.18 ± 0.2 ^f^

Values are represented as mean ± SD. Values of each parameter assigned identical letters do not vary significantly.

## Data Availability

Data is contained within the article.
